# Physical activity in the prevention of peripheral artery disease in the elderly

**DOI:** 10.3389/fphys.2014.00012

**Published:** 2014-03-03

**Authors:** Gabriele G. Schiattarella, Cinzia Perrino, Fabio Magliulo, Andreina Carbone, Antonio G. Bruno, Michele De Paulis, Antonio Sorropago, Roberto V. Corrado, Roberta Bottino, Giovanni Menafra, Raffaele Abete, Evelina Toscano, Giuseppe Giugliano, Bruno Trimarco, Giovanni Esposito

**Affiliations:** Department of Advanced Biomedical Sciences, University of Naples Federico IINaples, Italy

**Keywords:** aging, atherosclerosis, PAD, exercise, fitness, claudication

## Abstract

Aging is a well-known cardiovascular risk factor and cardiovascular diseases (CVD) are estimated to be the most common cause of death in the elderly. Peripheral arterial disease (PAD) represents an important clinical manifestation of CVD leading to increase morbidity and mortality, especially in elderly population. The correct management of PAD population includes the prevention of cardiovascular events and relief of symptoms, most commonly intermittent claudication. Progressive physical activity is an effective treatment to improve walking distance and to reduce mortality and cardiovascular events in patients with PAD, however the ability to effectively engage in physical activity often declines with increasing age. The maintenance and increase of reserve functional capacity are important concepts in the elderly population. Ultimately, the goal in participation of physical activity in the healthy elderly population is maintenance and development of physical functional reserve capacity. Therefore, for individuals suffering of PAD, appropriate physical activity in the form of supervised exercise may serve as a primary therapy. Although there are few direct comparisons of therapeutic exercise programs vs. pharmacological or surgical interventions, these increases in walking distance are greater than those reported for the most widely used agents for claudication, pentoxyphylline, and cilostazol. Despite a reduction in mortality and improvement of quality of life caused by physical activity in the PAD population, the molecular, cellular, and functional changes that occur during physical activity are not completely understood. Therefore, this review article aims at presenting an overview of recent established clinical and molecular findings addressing the role of physical activity on PAD in the older population.

## Peripheral arterial disease and the elderly

Peripheral arterial disease (PAD) is a manifestation of systemic atherosclerosis affecting 12–20% of men and women older than 55–65 years (Giugliano et al., 2012a,b,c,d; Schiattarella et al., 2012; Vu et al., 2012; Gargiulo et al., 2013a,b). It has been calculated that about 27 million people in Europe and United States suffer from this pathology, representing a relevant socio-economic problem in western countries (Brevetti and Chiariello, 2004; Giugliano et al., 2012a,b,c,d). Elderly populations have more severe forms of atherosclerosis with a higher prevalence of polidistrectual disease including carotid arteries (Ilardi et al., 2011; Gargiulo et al., 2013a,b) and abdominal aorta (Perrino et al., 2011a,b; Giugliano et al., 2012a,b,c,d, Schiattarella et al., 2013, and usually develop a higher grade of disability compared with younger people (Cacciatore et al., 2004). Large epidemiological studies have shown that PAD and an abnormal ankle-brachial index (ABI), in addition to be an important cause of disability in its symptomatic forms (intermittent claudication and critical limb ischemia), are associated with an elevated risk of developing severe cardiovascular disease in both symptomatic or asymptomatic patients (Suominen et al., 2008; Giugliano et al., 2012a,b,c,d; Schiano et al., 2012; Marsico et al., 2013). The prevalence of PAD increases with the age (Kroger et al., 2006), and advanced age is seen as a risk factor for cardiovascular disease (Perk et al., 2012).

## Physical activity and risk factor control

Physical activity, defined as any bodily movement produced by skeletal muscles that results in energy expenditure greater than at rest (Shephard and Balady, 1999), provides important benefits to physical and mental health of most people, and particularly older adults, producing long-term positive effects also in older people who already have established diseases and disabilities. Moreover, physical activity and exercise training, which must be structured, planned, and repetitive, are beneficial for the health of cardiovascular system. In particular, physical activity can reduce the onset of atherosclerosis, modulating cardiovascular risk factors (Thompson et al., 2003). A steady, moderate exercise such as long walks at a brisk pace is recommended for moderate exercise most days a week or even every day (Thompson et al., 2003).

Physical training exerts significant preventive actions against the development of PAD, also in the elderly. These effects are mainly, even though not exclusively, linked to a better control of atherosclerotic risk factors achievable with exercise. In particular, physical activity helps to treat many established atherosclerotic risk factors such as dyslipidemia, arterial hypertension, obesity, insulin resistance and glucose intolerance (Thompson et al., 2003). The combination of exercise and body weight loss leads to a decrease of circulating low density lipoprotein levels (LDL-C), and a concomitant rise in high density lipoprotein levels (HDL-C), and a dose-response relationship between the amount of exercise and HDL-C levels has been suggested (Drygas et al., 2000; Leon and Sanchez, 2001; Thompson et al., 2003). Greater increases in HDL-C might be found in subjects with hypertriglyceridemia (Thompson and Rader, 2001).

At least 44 randomized and controlled trials including 2764 patients have linked exercise training to lower blood pressure. Although exercise causes a decrease of arterial systo-diastolic pressure levels, the amount of such reduction depends on baseline blood pressure values (Fagard, 2001). Training from three to five times/week during 30–60 min sessions reduces blood pressure, particularly in hypertensive patients (Fagard, 2001). The extent of such effect is greater in mild hypertensive subjects who might benefit, at an early stage of the disease, from the physical activity alone, avoiding drug therapy.

Steady and regular physical activity is of crucial importance also in the prevention of insulin-resistance and glucose intolerance, producing a decrease in HbA1c of approximately 0.5–1% (Thompson et al., 2001). The Diabetes Prevention Program has demonstrated that physical activity and weight loss can help to prevent the onset of type II diabetes in high-risk subjects (Knowler et al., 2002). Exercise is also important to firstly gain and thereafter maintain body weight loss (Wing and Hill, 2001).

## Molecular mechanism of physical activity-induced prevention of atherosclerosis

Beyond a more strict control of atherosclerotic risk factors, physical exercise seems to exert “pleiotropic” healthy actions on the cardiovascular system (Perrino et al., 2011a,b) and, in particular, on structural and functional integrity of arterial vessels. Endothelial dysfunction is defined as a functional, and almost partially reversible alteration of endothelial cells, firstly resulting from impairment in nitric oxide (NO) availability (Virdis et al., 2010). NO exerts several homeostatic functions such as modulation of vascular tone, endogenous fibrinolysis and vascular inflammation (Virdis et al., 2010). Aging is associated with a progressive impairment of endothelial function in large epicardial arteries as well as in the coronary microcirculation (Versari et al., 2009; Gargiulo et al., 2013a,b). Advancing age progressively impairs the maximal forearm vasodilator response to acetylcholine, while the response to sodium nitroprusside, which is an endothelium-independent vasodilator, results minimally affected by aging (Taddei et al., 2001). Reduced NO availability observed in healthy subjects older than 70 years seems to be not responsive to L-Arginine, the initial substrate for NO synthase, differently from what observed in younger subjects (Taddei et al., 2001), suggesting that increased reactive oxygen species (ROS) generation in older subjects might significantly reduce endothelial-dependent vasodilation by inactivating NO. Also, a decreased expression of endothelial nitric oxide syntase (eNOS) and eNOS cofactor BH4 deficiency have been suggested to be as an additional mechanism contributing to the age-related decrease in NO production (Minamino et al., 2002). Not only decreased NO bioavaibility, but also increased levels of endothelium-derived vasocoacting factors such as Endotelin 1, Angiotensin II and thromboxane (TxA) are involved (Van Guilder et al., 2007; Virdis et al., 2010).

Other features of endothelial dysfunction beyond vascular tone dysregulation in the elderly include altered platelet aggregation, pro-thrombotic diathesis, vascular smooth muscle cell proliferation and migration, monocyte adhesion, adhesion molecule expression and impaired angiogenesis (Seals et al., 2008). Brachial artery flow-mediated dilatation has resulted to be inversely associated with intima-media thickness (IMT) (Juonala et al., 2004), while Rundek et al. have reported an association between endothelial dysfunction of the conduit artery and carotid plaque in a multi-ethnic population of elderly men and women (Rundek et al., 2006). Conversely, several evidences suggest that the beneficial role of physical activity on aging might be related to the amelioration of endothelial dysfunction. To address this issue, Taddei et al. recruited young and elder professional athletes and sedentary subjects. Among the elderly sedentary subjects, response to acetylcholine was significantly reduced compared to elderly athletes (Taddei et al., 2000). These data suggest that in healthy subjects physical activity may, at least partially, prevent age-related endothelial dysfunction, probably through an increase of NO availability, and in turn preventing the production of oxidative species. DeVan et al. also demonstrated that age-associated vascular endothelial dysfunction is exacerbated by impaired fasting plasma glucose, and that regular aerobic exercise prevents this effect (Devan et al., 2013). They found that among middle/old aged adults with impaired fasting glucose, brachial artery flow mediated dilation (FMD) was completely preserved in those subjects who regularly performed aerobic exercise when compared with their sedentary counter peers (Devan et al., 2013).

Physical activity also exerts benefits on the angiogenic function and in particular on endothelial progenitor cells (EPCs) number and function (Dimmeler and Vasa-Nicotera, 2003; Heiss et al., 2005). EPCs are a population of circulating cells, derived from bone marrow, with the ability to differentiate into endothelial cells. These cells have a critical role in the modulation of vascular homeostasis, being able to enhance angiogenesis, to promote vascular repair and endothelial cells turn-over, to improve endothelial function, and finally to inhibit atherosclerosis (Asahara et al., 1997; Hill et al., 2003; Werner et al., 2003). Cardiovascular diseases and aging are associated with reduced numbers and function of circulating EPCs (Hill et al., 2003; Scheubel et al., 2003). However, in patients with cardiovascular risk factors and CVD, aerobic activity enhances EPCs number and function. In patients with stable coronary artery disease, exercise training for 28 days led to a significant increase in circulating EPCs and reduced EPCs apoptosis (Laufs et al., 2004). The observed increase in EPCs may contribute to the favorable effects of physical exercise on cardiovascular health. Moreover, Brixius et al. demonstrate that endurance training stimulate neo-angiogenesis by inhibiting the anti-angiogenic signaling of an endogenous 20-kDa C-terminal fragment peptide derived from type XVIII collagen named endostatin (Brixius et al., 2008). Endostatin has been found, for the first time, in the secretion medium of non-metastasizing mouse cells from a hemangioendothelioma cell line in 1997 and was subsequently found in humans (O'Reilly et al., 1997; Standker et al., 1997). Coagulation cascade seems to be involved into the genesis of this peptide. Endostatin action depends on down-regulation of TNF-α/NF-kB cascade signaling as well as coagulation and adhesion (Abdollahi et al., 2004). In the above cited study, a significant decrease in plasma level of endostatin was observed in 50–60 years aged obese men subjected to two different training program (running or cycling three times/week) when compared with same-aged sedentary controls (Brixius et al., 2008).

## Physical activity prevent peripheral atherosclerosis

As described above, physical activity allows a better control of atherosclerotic risk factors (lower blood pressure, increased HDL-C, increased glycemic control etc.) and preserves vessel integrity by restoring endothelial function. Another mechanism potentially involved into exercise induced prevention from vascular remodeling regards a decrease in vascular tone, due to a slighter sympathetic system activity (Dinenno et al., 2000).

There is an adequate number of observations concerning exercise and prevention of atherosclerotic lesions development into peripheral vessels. Atherosclerosis can begin in early life (Tuzcu et al., 2001), with impairment of endothelial function (Celermajer et al., 1992), followed by a progressive remodeling of the arterial wall (De Groot et al., 2008). Plaque evolution is prolonged and occult, particularly in the first phases. These considerations emphasize the potential utility of vascular ultrasound to detect arterial wall thickness and to measure IMT which is a validated surrogate marker for atherosclerosis (De Groot et al., 2008). Common carotid IMT is associated with increased risk for adverse cardiovascular events such as myocardial infarction and stroke (Chambless et al., 1996; Bots et al., 1997) and, also, for the future development of peripheral arterial disease (Wattanakit et al., 2005). Thickening of the femoral wall correlates with measures of peripheral atherosclerotic disease, such as the ankle–brachial index (Suurkula et al., 1996). An augmented femoral artery IMT also strongly relates to traditional cardiovascular risk factors, such as blood pressure, waist circumference, cholesterol, insulin, and smoking status (Paul et al., 2005, 2010). Advanced age is also associated with vascular thickening (Thijssen et al., 2012). Combined studies concerning carotid IMT and self-reported physical practice—including a total of >28000 subjects—found an inverse relationship between these variables (Thijssen et al., 2012). Moreover, an higher *a priori* physical practice level is related to fewer 3- or 6-years increases in carotid IMT (Nordstrom et al., 2003); the inverse relationship between exercise and carotid artery atherosclerosis was observed in men, but not in women (Thijssen et al., 2012). Cross-sectional studies in middle-aged and older humans have reported that low fitness correlates independently with increased carotid IMT (Rauramaa et al., 1995) and with the presence of carotid plaque (Lee et al., 2009). A more pronounced effect has been observed into patients with hypertension, diabetes and dyslipidemia (Thijssen et al., 2012). Gando et al. have found an independent inverse correlation between carotid IMT, lumen diameter, wall mass and VO2 peak into middle aged men performing cardiorespiratory fitness (Gando et al., 2011). So, physical exercise seems to prevent subclinical atherosclerosis. Interestingly, exercise training seems to exert more pronounced effects on large peripheral arteries than on epi-aortic vessels (Thijssen et al., 2012). Modification of carotid wall thickness probably require more intense or prolonged exercise exposure (Thijssen et al., 2012).

## Exercise and treatment of symptomatic PAD

Progressive physical activity is also used as an effective treatment to improve walking distance in patients with PAD. Supervised exercise may be useful as primary therapy for many patients with claudication. A meta-analysis of 21 studies showed an increase in the distance to onset of claudication pain of 179% with exercise therapy (Gardner and Poehlman, 1995). The greatest improvement in pain distances occurred with the following exercise program: duration greater than 30 min per session, frequency of at least three sessions per week, walking used as the mode of exercise, use of near-maximal pain during training as claudication pain end point, and program length of greater than 6 months (Gardner and Poehlman, 1995). These increases are greater than those obtained with widely used drugs such as pentoxifylline and cilostazol (Gardner and Poehlman, 1995). As a consequence, the European Society of Cardiology proposes walking training as first-step therapy for claudicant patients before percutaneous or surgical options (Tendera et al., 2011). Supervised training seems to be significantly better in terms of efficacy, measured in walking meter improvements, than programs of not-supervised exercise therapy (Bendermacher et al., 2006). Furthermore other important issues should be underlined: drugs which have been proposed for the treatment of PAD are not disease-modifiers agents. For example, cilostazol, naftidrofuryl, and pentoxifylline are arterial vasodilatant agents, while l-propionylcarnitine is an amino acid naturally produced in the body whose biological function is to promote fat acid β-oxidation by enabling acil-CoA transport into mitochondria- so, its action is related to an improvement of metabolism into ischemic skeletal muscles. None of these drugs acts preserving vascular integrity, such as exercise training, as widely described above. As an exception, statin therapy has been associated with a large number of “pleiotropic” effects favorably affecting endothelium function and vessel structure; their use has been demonstrated to be safe and effective in the elderly (Schiattarella et al., 2012) and is, nowadays, a cornerstone in the treatment of PAD (Gargiulo et al., 2012). While the superiority of physical training on pharmacological strategy in the treatment of PAD has been clearly assessed, their actions might be complementary, because pharmacological compounds may favor patient compliance to exercise. Obviously, there are some limitations to training therapy, especially in older subjects, including the presence of muscular, articular, and/or neurological diseases, and also cardiac and/or pulmonary diseases can be limiting factors.

## Conclusions

PAD is a widespread cause of disability in the elderly population in the Western world. Preventing the classic risk factors for atherosclerosis, through a balanced diet and moderate and steady physical activity together with classic pharmacological therapy might decrease the incidence of this disease. The molecular mechanisms by which this effect is achieved are not completely understood, but various mediators are involved in this process, including NO, EPCs, and growth factors such as VEGF. Given the fundamental importance of anti-oxidant properties of physical practice, it is conceivable that a contemporary anti-oxidant rich diet might enhance the outcome. In these recent years nutraceuticals have gained a growing interest in geriatric medicine since they have a large number of beneficial effects and provide an efficient replace of micronutrient deficit frequently found in elderly people. Their actions, with a particular concern to cardiovascular protection, are summarized in Table 1. Among, these, the extracts of Ginkgo biloba, a Chinese tree, which are rich in flavonoid glycosides, poliphenols, and terpenoids, have been reported to improve minimal walking distance to pain in claudicant people; however, two recent trials did not show a superiority of Gingko biloba administration compared to exercise alone (Wang et al., 2007; Gardner et al., 2008).

**Table 1:** **Nutraceutical and prevention of cardiovascular disease**.

**Active compounds**	**Dosage**	**Reported results**
Vitamin C	800 mg daily	Antioxidant properties
		Preserved endothelial function
		Poor evidences in human studies (no reduction in major CV events)
Vitamin E	600 mg daily	Antioxidant properties
		Significant reduction in urinary tromboxane concentration
		No evidences for routine use
Calcium citrate + vitamin D_3_	500 mg + 700 IU daily	In healthy adults supplementation may attenuate glycemia increase
		Prevention of ostheoporosis
Flavonoids (such as quercetin, myricetin, naringemin, hesperetin)	Various dosages	Risk reduction of diabetes mellitus
		Decreased incidence of ischemic heart disease and mortality after AMI
		Lower incidence of CV death and morbidity
Omega-3-Fatty acid	1000 mg tid	Decrease blood triglycerides levels
		Prevent atherosclerosis
		Inconsistent data in secondary prevention of CV diseases
Chromium	300 μg daily of elemental Cr (III)	Mean fasting glucose levels lowered
Zinc sulfate 200 mg daily	200 mg daily	Modest benefit of treatment
α-lipoic acid	600 mg/day	Increase in insulin-resistance
Resveratol	50 mg/day	Block endothelial senescence
		Lower serum lipids and arterial pressure
		Decrease inflammation

CV, cardiovascular; IU, international units; AMI, acute myocardial infarction; tid, three time a day.

Physical exercise exerts a huge number of beneficial functions on vascular function; however, it must also be underlined that maintaining an adequate level of functional capacity in the elderly also preserves the integrity of all other organs and systems such as osteo-articular, muscular respiratory and nervous system, with also positive outcomes on psychophysical wellness. Sedentary lifestyle in older subjects is associated with many adverse complications such as osteoporosis, degenerative arthritis and depression, which conversely decrease functional reserves and lead to progressive immobilization, muscular atrophy and, eventually, till to severe disability and bedrittening (Cacciatore et al., 2004). Obviously, inactivity is a significant risk factor for atherosclerosis and cardiovascular diseases. So, while it is not hard to realize that sedentary habitus may produce a perilous circle, physical exercise preserves cardiovascular system from atherosclerotic disease, and other organic systems from negative consequences related to aging.

### Conflict of interest statement

The authors declare that the research was conducted in the absence of any commercial or financial relationships that could be construed as a potential conflict of interest.
